# A Hybrid Approach for Heart Disease Diagnosis and Prediction Using Machine Learning Techniques

**DOI:** 10.1007/978-3-030-51517-1_26

**Published:** 2020-05-31

**Authors:** Fatma Zahra Abdeldjouad, Menaouer Brahami, Nada Matta

**Affiliations:** 8 Digital Research Centre of Sfax, Sfax, Tunisia; 9 Institut Mines-Télécom, CNRS, Paris, France; 10 Université de Sherbrooke, Sherbrooke, QC Canada; 11 Digital Research Centre of Sfax, Sfax, Tunisia; 12 University of Sfax, Sfax, Tunisia; 13 National Polytechnic School of Oran - Maurice Audin, Oran, Algeria; 14 University of Technology of Troyes, Troyes, France

**Keywords:** Machine learning, Data mining, Healthcare informatics, Heart disease, Classification, Prediction models, Medical decision support system

## Abstract

Heart disease is considered as one of the major causes of death throughout the world. It cannot be easily predicted by the medical practitioners as it is a difficult task which demands expertise and higher knowledge for prediction. Currently, the recent development in medical supportive technologies based on data mining, machine learning plays an important role in predicting cardiovascular diseases. In this paper, we propose a new hybrid approach to predict cardiovascular disease using different machine learning techniques such as Logistic Regression (LR), Adaptive Boosting (AdaBoostM1), Multi-Objective Evolutionary Fuzzy Classifier (MOEFC), Fuzzy Unordered Rule Induction (FURIA), Genetic Fuzzy System-LogitBoost (GFS-LB) and Fuzzy Hybrid Genetic Based Machine Learning (FH-GBML). For this purpose, the accuracy and results of each classifier have been compared, with the best classifier chosen for a more accurate cardiovascular prediction. With this objective, we use two free software (Weka and Keel).

## Introduction

One of the most common reasons of death in Algeria or other Maghreb countries is chronic disease. Nevertheless, chronic disease is a vital issue to be fixed for a healthy human life. More recently, Cardiovascular Disease (CVD) is the leading cause of death for both men and women globally. Though real-life consultants can be able to predict the disease with an enormous number of tests and requiring a huge processing time, sometimes, their prediction may be incorrect because of lack of skilled knowledge [1]. Meanwhile, the introduction of artificial intelligence and machine learning has helped to extract relevant data from large databases which are available in hospitals to make a good decision. It involves data mining techniques to analyze medical data [2]. For this reason, data mining has gained popularity due to its tools with the potential to identify trends within data and turn them into knowledge that could serve as the strong basis for the analysis [3]. To that end, the key issue in the field of CVD prevention is to give an accurate prediction of whether a person is probable to have this disease. Motivated by the growing mortality of CVD patients every year and the accessibility to a huge amount of patient data from which to obtain valuable knowledge, we found it useful to use data mining methods for assisting healthcare professionals in the diagnosis of CVD. The objective of this research work is not to replace the specialist physician, but to assist the doctor in obtaining an alternative opinion and its various feasibility in critical situations.

The rest of this paper is organized as follows. Section 2 describes the literature review. Section 3 presents the proposed approach used for predicting heart disease. Experimental results are analyzed in Sect. 4 and Conclusion and References are given in Sect. 5 and 6.

## Literature Review

In previous studies, researchers expressed their efforts in finding the best model for predicting cardiovascular disease. In the meantime, various studies give only a glimpse into predicting heart disease using machine learning techniques and fuzzy logic systems. This section explores the research works that are related to the proposed approach. A machine learning model has been proposed in [2] by combining five different algorithms. In fact, the integration of the machine learning model with medical information systems would be useful to predict the Heart Failure (HF) or any other disease using the live data collected from patients. A new hybrid approach for heart disease prediction that combines all techniques into one single algorithm has been proposed in [4]. The result confirms that accurate diagnosis can be made using a combined model from all techniques. An “Optimal Multi-Nominal Logistic Regression (OMLR) algorithm has been proposed in [5] and is used to train the data set for heart disease. Experiments are conducted on the dataset of UCI heart disease and the results show 92% accuracy in the detection of heart severity. The Fast Correlation-Based Feature Selection (FCBF) method has been exploited in [6], to filter redundant features in order to improve the quality of heart disease classification. Then, the authors performed a classification based on different algorithms such as K-Nearest Neighbour, Support Vector Machine, Random Forest and a Multilayer Perception optimized by Particle Swarm Optimization (PSO) combined with Ant Colony Optimization (ACO) approaches. A predictive model for heart disease diagnosis using a fuzzy rule-based approach with decision tree has been proposed in [7]. In this study, the authors have obtained the accuracy of 88% which is statistically significant for diagnosing the heart disease patient and also outperforms some of the existing methods. A new method namely Hybrid Differential Evolution based Fuzzy Neural Network (HDEFNN) which can predict the heart disease occurrence fastly and accurately has been proposed in [8]. The performance of this method in terms of accurate diagnosis of heart disease is attained by improving the initial weight updating of a neural network which is done by introducing the genetic algorithm. The genetic algorithm can select the most optimal weight values for the hidden layers of the neural network. A neuro-fuzzy genetic approach has been proposed in [9], to predict chances of cardiovascular disease. The proposed approach also helps to make the system more accurate and efficient with the help of a genetic algorithm.

## Proposed Approach

(See Fig. 1).

**Fig. 1.**
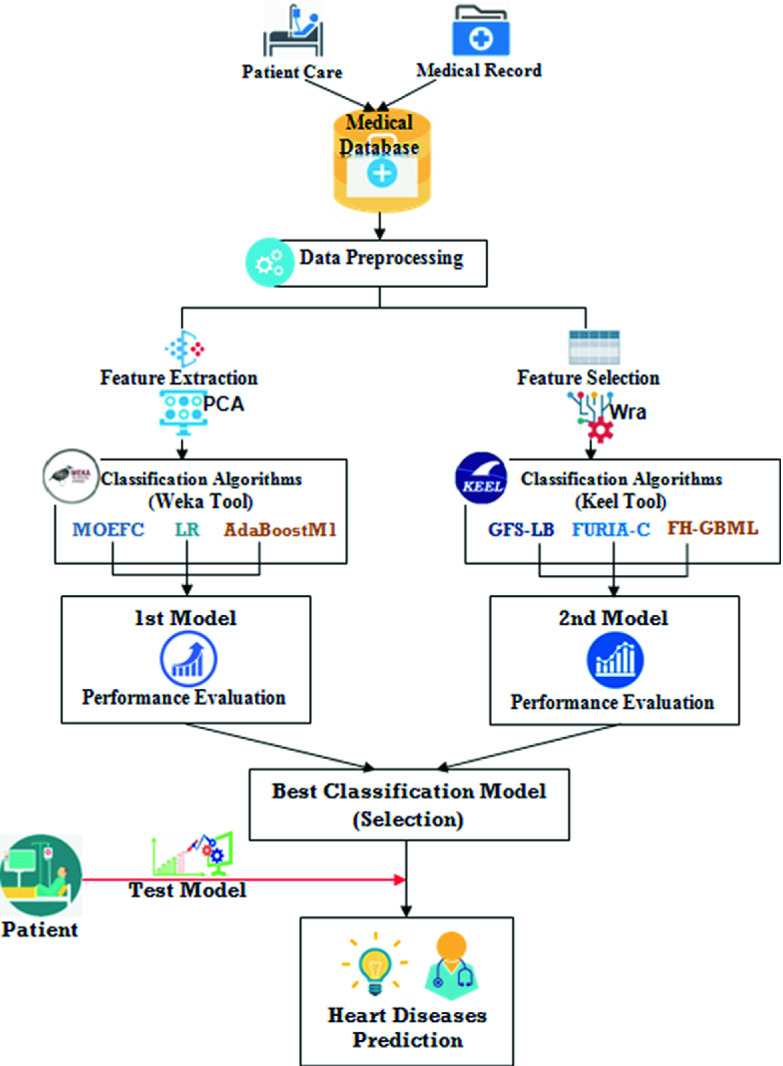
General architecture of the proposed approach

### Description of the Dataset and Attributes

The dataset used in this article is taken from the UCI Repository Of Machine Learning Databases^1^. Formally, it is named Heart Disease Dataset. The Cleveland (Cleveland Clinic Foundation) database was selected for this research because it is a commonly used database for machine learning researchers with comprehensive and complete records. In this field, the dataset is a collection of medical analytical reports with a total of 303 records with 14 medical features. The various features and their description are shown in Table 1. Besides, the categorical feature “Class” contains whether a patient has a presence or absence of heart disease. Its original values 1, 2, 3 and 4 were transformed in one that is the presence (1) of heart disease.

**Table 1.** UCI dataset attributes detailed information

Num.	Code	Feature	Type	Description
1	Age	Age	Continuous	Age in years
2	Sex	Sex	Discrete	sex (1 = male; 0 = female)
3	Cp	Chest pain type	Discrete	1 = typical angina; 2 = atypical angina; 3 = non-angina pain; 4 = asymptomatic
4	Trestbps	Resting boold pressure (mg)	Continuous	At the time of admission in hospital [94, 200]
5	Chol	Serum cholesterol (mg/dl)	Continuous	Multiple values between [Minimum Chol: 126, Maximum Chol: 564]
6	Fbs	Fasting bood sugar > 120 mg/dl	Discrete	1 = yes; 0 = no
7	Restecg	Resting electrocardiographic results	Discrete	0 = normal; 1 = ST-T wave abnormal; 2 = left ventricular hypertrophy
8	Thalach	Maximum heart rate achieved	Continuous	Maximum heart rate achieved [71, 202]
9	Exang	Exercise induced angina	Discrete	1 = yes; 0 = no
10	Oldpeak	ST depression induced by exercise relative to rest	Continuous	Multiple real number values between 0 and 6.2.
11	Slope	The slope of the peak exercise ST segment	Discrete	1 = upsloping; 2 = flat; 3 = downsloping
12	Ca	Number of major vessels (0–3) colored by fluoroscopy	Discrete	Number of major vessels coloured by fluoroscopy (values 0–3)
13	Thal	Exercise thallium scintigraphy	Discrete	3 = normal; 6 = fixed defect; 7 = reversible defect
14	Class (Target)	The predicted attribute	Discrete	0 = no presence; 1 = presence

### Data Pre-processing

In medical informatics, the diagnosis of diseases becomes quicker and easier if data is free from missing, redundant and irrelevant data. In this study and after collection of various records, we begin the preprocessing process. The dataset contains a total of 303 patients records, where 7 records are with some missing values. Those 7 records have been removed from the dataset and the remaining 296 records are used in the process.

### Feature Selection

Feature selection is a process of selecting a relevant feature of original features according to definite condition. Further, feature collection algorithms intended with different evaluation criteria mostly fall into three categories: the filter, wrapper, and hybrid models [10]. In our work, we used only the wrapper method under Keel tool. As per our objective, from among the 14 attributes of the dataset, two attributes pertaining to age and sex are used to identify the personal information of the patient. The remaining 12 attributes are considered important as they contain vital clinical records.

### Feature Extraction

Feature extraction is a process that extracts a subset of new features from the original set by means of some functional mapping. In order to meet the goal of the work, we used PCA as one of the most widely used dimensionality reduction technique for the medical applications under Weka tool, where the extracted information is represented by a set of new variables, termed components or features. With PCA, we reduced the attributes number to 6 which contributes more towards the diagnosis of the CVD.

### Classification Algorithms

Under Weka tool, different predictive algorithms were chosen to build the first model, namely: Multi-Objective Evolutionary Fuzzy Classifier (MOEFC), Logistic Regression (LR), Adaptive Boosting (AdaBoostM1), while Genetic Fuzzy System-LogitBoost (GFS-LB), Fuzzy Unordered Rule Induction Algorithm (FURIA) and Fuzzy Hybrid Genetic Based Machine Learning (FH-GBML) were used under Keel tool to build the second model. Therefore, we selected the best model in order to achieve the highest possible performance on medical datasets and allow effective data classification.

### Test Model

In the second stage, we tested our selected model only when the model is completely trained. Its accuracy on the test data gives a realistic estimate of the model performance on completely unseen patient data and confirms the actual predictive power of the model.

## Experimental Results

In this paper, the experimental effects of the cardiovascular diseases’ diagnosis and the following algorithms LR, AdaBoostM1, MOEFC, FURIA, GFS-LB and FH-GBML are examined in this phase with the use of Keel and Weka tools. Meanwhile, machine learning algorithm efficiency is derived using values like True Positive (TP), True Negative (TN), False Positive (FP) and False Negative (FN). These measures are used for the calculation of the sensitivity, specificity, accuracy and error rate.

1 $$ {\text{Sensitivity}}\;\left( {\text{Recall}} \right)\;{\text{or}}\;{\text{True}}\;{\text{positive}}\;{\text{rate}}\;\left( {\text{TPR}} \right)\, = \,{\text{TP}}/\left( {{\text{TP}}\, + \,{\text{FN}}} \right). $$

2 $$ {\text{Specificity}}\, = \,{\text{TN}}/\left( {{\text{TN}}\, + \,{\text{FP}}} \right) $$

3 $$ {\text{Accuracy}}\;\left( {\text{ACC}} \right)\, = \,\left( {{\text{TP}}\, + \,{\text{TN}}} \right)/\left( {{\text{TP}}\, + \,{\text{TN}}\, + \,{\text{FP}}\, + \,{\text{FN}}} \right). $$

4 $$ {\text{Error}}\;{\text{rate}}\, = \,\left( {{\text{FP}}\, + \,{\text{FN}}} \right)/\left( {{\text{P}}\, + \,{\text{N}}} \right). $$

### Evaluation of Results

#### Setting up the Experiment under WEKA Software.

In our experiment, the problem has been transformed into binary classification with 0 presents absence and 1 presence of heart disease. For this, Table 2 shows the results obtained by binary classification and 10-fold cross-validation. The highest accuracy 80.20 is gained by majority voting, while LR obtained lowest accuracy and AdaBoostM1 has the highest accuracy when applied without ensemble.

**Table 2.** Multi-class classification results by 10-fold cross-validation

Algorithm	Sensitivity	Specificity	Accuracy
MOEFC	79.96	75.44	79.42
LR	78.22	71.34	78.77
AdaBoostM1	80.11	75.40	80.01
Vote	84.76	74.82	80.20

#### Setting Up the Experiment under KEEL Software.

Our purpose is to make a comparison of three methods that belong to different ML techniques. In this step, we have used a GFS-LogitBoost-C classifier with a previous pre-processing stage of prototype selection guided by a Generational Genetic Algorithm for Feature Selection (GGA-FS) model. We have also used a FURIA classifier with a previous preprocessing stage of replacing missing values guided by a KNN-MV (K-Nearest Neighbor Imputation) algorithm as well as prototype feature selection guided by SSGA-Integer-knn-FS (Steady-state GA with integer coding scheme for wrapper feature selection with K-NN) and an FH-GBML that uses a Generational Genetic Algorithm for Feature Selection (GGA-FS). After the models are trained, the instances of the dataset are classified according to the training and test files. These results are the inputs for the visualization and test modules. The module Vis-Clas-Tabular receives these results as inputs and generates output files with several performance metrics computed from them, such as confusion matrices for each method. There is also another type of results flow which interconnects each possible pair of methods with a test module. In this case, the test module used is the signed-rank Wilcoxon non-parametrical procedure Clas-Wilcoxon-ST which compares two samples of results. The experiment establishes a pair-wise statistical comparison of the three methods. Once the experiment has been run we can reach results shown in Table 3 and Table 4.

**Table 3.** Performance of the KEEL model - training datasets

Evaluation criteria	FURIA-C	GFS-LogitBoost-C	FH-GBML-C
Sensitivity	88.62	94.99	87.47
Specificity	76.26	93.20	78.66
Error rate	0.17	0.06	0.17
Accuracy	82.95	94.17	83.44

**Table 4.** Performance of the KEEL model - testing datasets

Evaluation criteria	FURIA-C	GFS-LogitBoost-C	FH-GBML-C
Sensitivity	84.76	80.49	82.82
Specificity	74.82	80.58	74.26
Error rate	0.20	0.19	0.21
Accuracy	80.20	80.53	78.93

## Conclusion

Efficient classification of healthcare dataset is a major machine learning problem then and now. Diagnosis, Prediction of cardiovascular diseases and the precision of results can be improved if relationships and patterns from these complex healthcare datasets are extracted efficiently. This paper analyses some of the different classification algorithms like Logistic Regression (LR), Adaptive Boosting (AdaBoostM1), Multi-Objective Evolutionary Fuzzy Classifier (MOEFC), Fuzzy Unordered Rule Induction (FURIA), Genetic Fuzzy System-LogitBoost (GFS-LB) and Fuzzy Hybrid Genetic Based Machine Learning (FH-GBML). The performance evaluation of these algorithms is done based on Accuracy, Sensitivity, Specificity and Error rate using WEKA and KEEL tools.

## Footnotes

1 Repository Of Machine Learning (UCI Databases). Heart Disease Data Set. [Online]. Available: https://archive.ics.uci.edu/ml/machine-learning-databases/heart-disease/heart-disease.names [Accessed: June 20, 2019].
